# Normal Anatomy Mimicking an Abdominal Aortic Dissection

**DOI:** 10.24908/pocusj.v11i01.20821

**Published:** 2026-04-22

**Authors:** Olivia Klee, Julia Buechler, Molly Fears, Caroline Gosser, Jeffery Baker, Kahra Nix

**Affiliations:** 1 School of Medicine, University of Louisville, Louisville, KY, USA; 2 Department of Emergency Medicine, School of Medicine, University of Louisville, Louisville, KY, USA

**Keywords:** Point-of-care ultrasound, POCUS, Abdominal Aorta, Aortic Dissection

## Abstract

Point-of-care ultrasound is often taught to learners at the bedside using standardized patients. Just as in clinical practice, there is the potential for coming across incidental findings or even pathology mimics. We describe a series of four cases where normal anatomy mimicked an abdominal aortic dissection. We clarify normal anatomy and probe maneuvers to prevent from mistaking this from pathology.

This article is a corrigendum to: Klee O, Buechler J, Fears M, Gosser C, Nix K. A Point of Care Ultrasound (POCUS) Artifact Mimicking an Aortic Dissection: A Case Series. POCUS J. 2025 Apr 15;10(1):88-91. doi: https://doi.org/10.24908/pocusj.v10i01.18498

In the original article, the authors highlighted a key POCUS mimic of abdominal aortic dissection in a series of young patients who were later referred for diagnostic radiology imaging and found to have normal aortas. This was attributed to artifact in the case series, however, it was later determined that this “pseudo-dissection” finding on POCUS is a result of how the echogenic line of a normal diaphragmatic crus tricks the POCUS user into interpreting the normal aortic wall as a possible dissection flap. This corrigendum provides a corrected interpretation of the original findings.

## Introduction

Point of care ultrasound (POCUS) is evaluated along with other knowledge, skills, attitudes, and attributes in the Emergency Medicine Milestones, as defined by the American College of Graduate Medical Education [1]. In a policy statement, the American College of Emergency Physicians acknowledged POCUS as a fundamental part of Emergency Medicine (EM) training [2]. In order to achieve these goals, EM residents have discrete and longitudinal POCUS training. Often, medical students are included in bedside teaching both in the operator role and as standardized patients. When acting as a standardized patient, verbal consent is obtained. The potential for incidental findings is acknowledged as a possibility with a clear plan for next steps [3]. In this case series, we describe normal anatomy that mimics a dissection involving the abdominal aorta that was found on a young, healthy, thin female medical student who was acting as a standardized patient. This same abdominal aortic dissection mimic was subsequently seen on three additional young, healthy, thin, female medical students.

## Methods

Each of the standardized patients in this case series provided written, informed consent for the POCUS images to be obtained after a full explanation was provided. The abdominal aortas from four standardized patients were evaluated using a Sonosite curvilinear (5-1 MHz) (Sonosite, Bothwell, WA, USA) or a Mindray curvilinear (C6-1s) transducer (Mindray, Mahwah, NJ, USA) by placing the probe in the sagittal and transverse planes along the abdominal aorta. Clips and stills of both the sagittal and transverse views of the abdominal aorta were recorded. Additional clips with color Doppler were obtained on the initial standardized patient and one other. This case series was completed after the University of Louisville Institutional Review Board determined it was exempt. Each standardized patient consented to this case series.

## Case Presentation and Results

### Case 1

**Figure 1.**
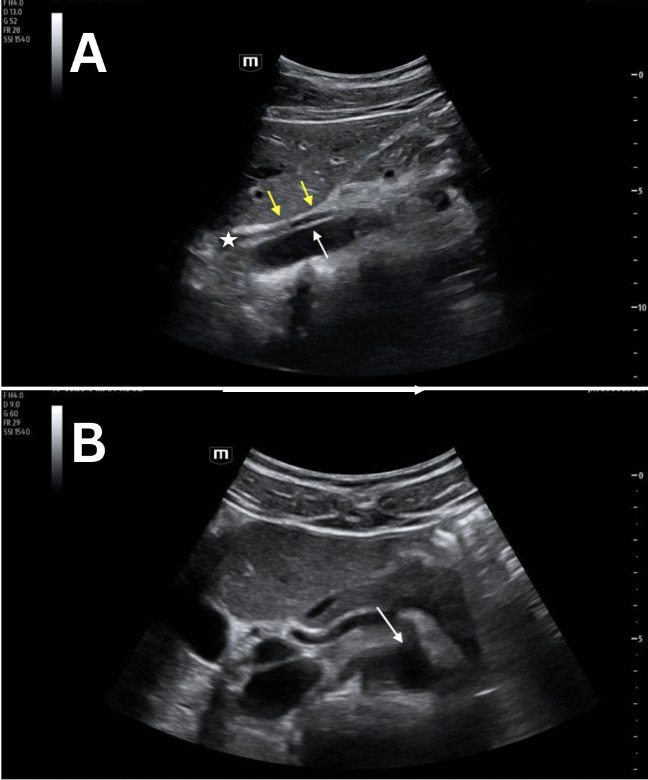
Grayscale point of care ultrasound (POCUS) images of a 22-year-old female standardized patient showing (A) sagittal view with two neighboring linear, hyperechoic structures (double yellow arrow and white arrow) and the intervening, hypoechoic crus of the diaphragm (white star) and (B) transverse view with a normal, anechoic lumen of the abdominal aorta (white arrow).

A POCUS examination of the abdominal aorta was performed on a 22-year-old medical student acting as a standardized patient. Grayscale sagittal-plane images on POCUS showed the abdominal aorta but did not clearly distinguish which of two neighboring structures represented the true aortic wall (Figure 1, Supplementary Material S1). The transverse view showed expected anatomy with no overt pathology.

### Case 2

**Figure 2.**
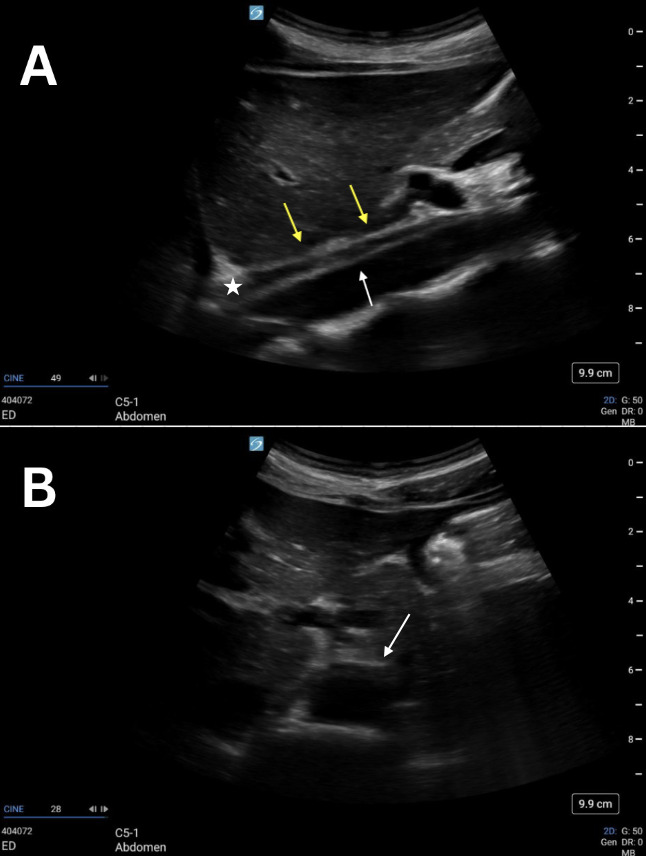
Grayscale point of care ultrasound (POCUS) images of a 25-year-old female standardized patient showing (A) sagittal view with two neighboring linear, hyperechoic structures (double yellow arrow and white arrow) and the intervening hypoechoic crus of the diaphragm (white star) and (B) transverse view with a normal, anechoic lumen of the abdominal aorta (white arrow).

A POCUS examination of the abdominal aorta was performed on a 25-year-old medical student acting as a standardized patient. Grayscale sagittal-plane images on POCUS showed the abdominal aorta but did not distinguish which of two neighboring structures represented the true aortic wall (Figure 2, Supplementary Material S2). The transverse view showed expected anatomy with no overt pathology.

### Case 3

**Figure 3.**
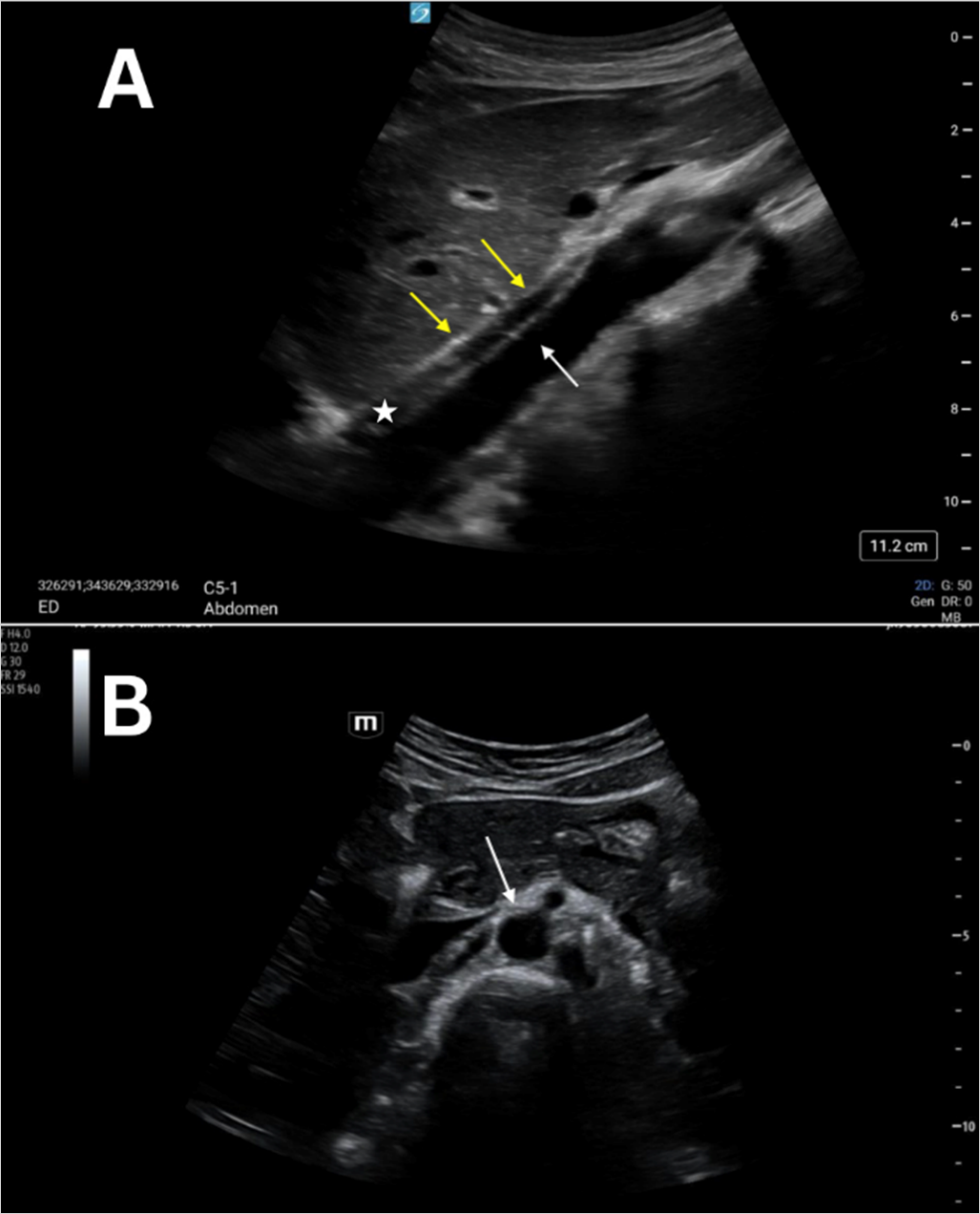
Grayscale point of care ultrasound (POCUS) images of a 27-year-old female standardized patient showing (A) sagittal view with two neighboring linear, hyperechoic structures (double yellow arrow and white arrow) and the intervening hypoechoic crus of the diaphragm (white star) and (B) transverse view with a normal, anechoic lumen of the abdominal aorta (white arrow).

A POCUS examination of the abdominal aorta was performed on a 27-year-old medical student acting as a standardized patient. Grayscale sagittal-plane images on POCUS showed the abdominal aorta but did not distinguish which of two neighboring structures represented the true aortic wall (Figure 3, Supplementary Material S3). The transverse view showed expected anatomy with no overt pathology.

### Case 4

**Figure 4.**
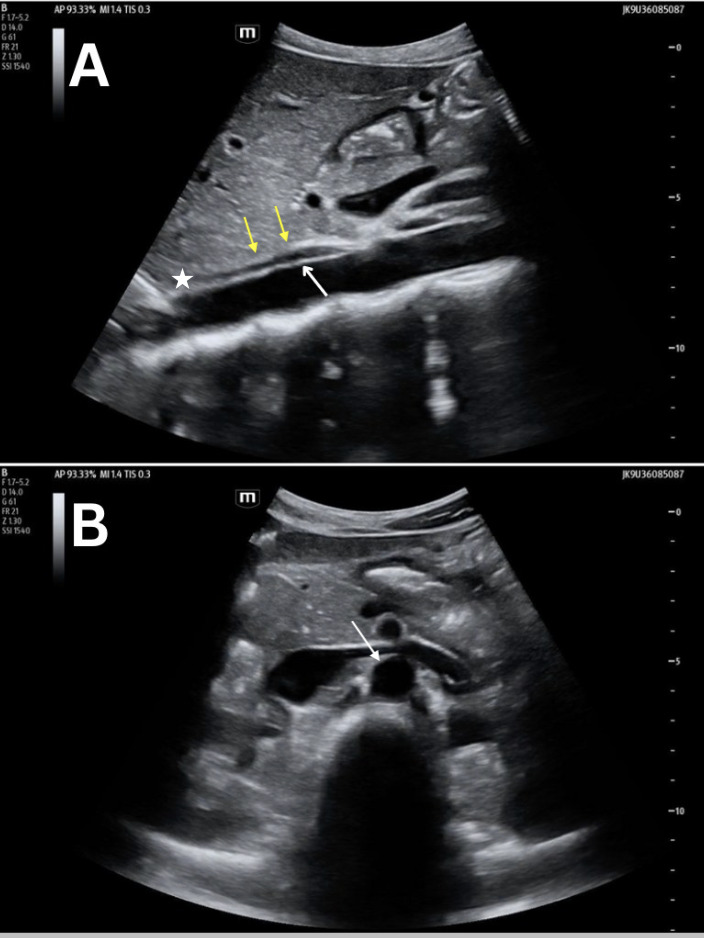
Grayscale point of care ultrasound (POCUS) images of a 25-year-old female standardized patient showing (A) sagittal view with two neighboring linear, hyperechoic structures (double yellow arrow and white arrow) and the intervening hypoechoic crus of the diaphragm (white star) and (B) transverse view with a normal, anechoic lumen of the abdominal aorta (white arrow).

A POCUS examination of the abdominal aorta was performed on a 25-year-old medical student acting as a standardized patient. Grayscale sagittal-plane images on POCUS showed the abdominal aorta but did not distinguish which of two neighboring structures represented the true aortic wall (Figure 4, Supplementary Material S4). The transverse view showed expected anatomy with no overt pathology.

### Additional Images

**Figure 5.**
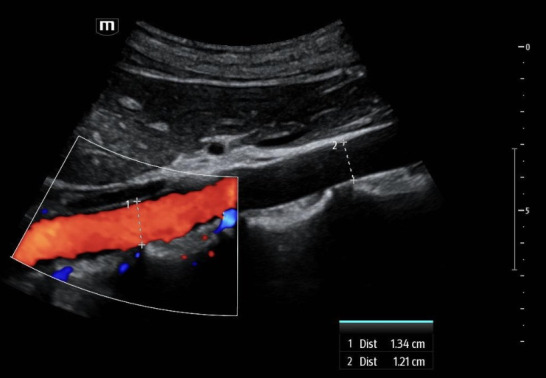
Point of care ultrasound (POCUS) images with color Doppler of a 22-year-old female standardized patient showing a sagittal view of the abdominal aorta. Color flow clarifies which of two neighboring linear hyperechoic structures is the true wall of the abdominal aorta.

**Figure 6.**
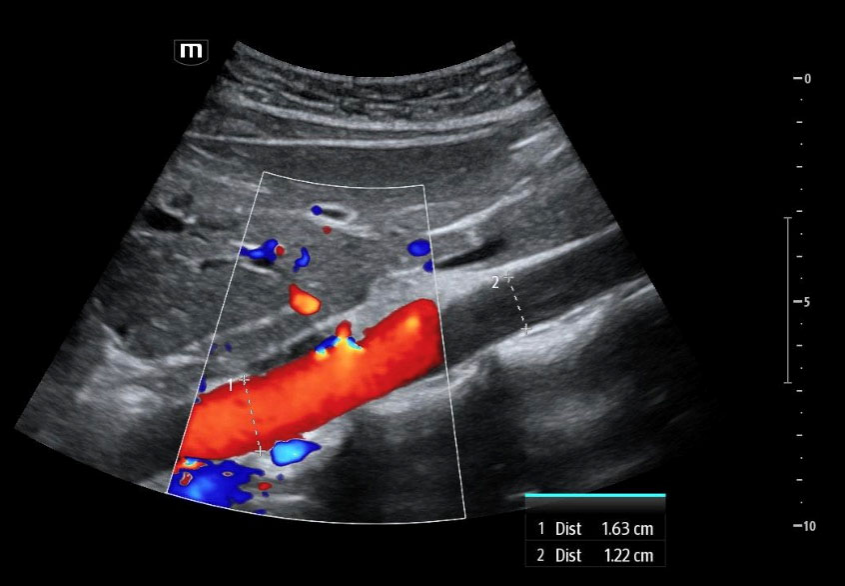
Point of care ultrasound (POCUS) images with color Doppler of a 27-year-old female standardized patient showing a sagittal view of the abdominal aorta. Color flow clarifies which of two neighboring linear hyperechoic structures is the true wall of the abdominal aorta.

Additional still images of the abdominal aorta in the sagittal plane with color Doppler were obtained from the standardized patients from Case 1 (Figure 5) and Case 3 (Figure 6), including measurements of the filled lumen along the visualized aorta. Within a margin of error, the measurements of the aorta's anterior-posterior dimension at two locations were congruent. Lastly, additional clips surveying the abdominal aorta from proximal to distal in the transverse plane were obtained from the standardized patients in Case 1 (Supplementary Material S5) and Case 3 (Supplementary Material S6).

## Discussion

POCUS mimics and artifacts are frequently encountered in both clinical and educational settings. Case 1 showed a sagittal view of the aorta that was confused for a possible and unexpected abdominal aortic dissection. This asymptomatic, healthy, standardized patient was sent for a radiology-performed ultrasound of her abdomen that confirmed her abdominal aorta as normal. Artifacts within vessels are known to occur as a result of mirroring and reverberation and can mimic both dissection and thrombus [4–7]. These resolve with rotation and/or translation of the probe. Our initial proposed etiology was that this was side-lobe artifact, which is known to occur within vessels as a result of the appearance of an echogenic structure that did not actually originate from within the vessel. As with other examples of side-lobe artifact, the linear, hyperechoic structure in these four cases disappeared when rotating the probe from the sagittal to the transverse plane (Figures 1-4).

However, there are multiple findings that point away from artifact as the explanation and toward normal anatomy that can mimic a dissection. Dissection flaps often undulate, and these did not. Also, color Doppler revealed the true wall (Figures 5 and 6) as there was continuous, uniform flow that filled the vessel lumen. A true dissection should exist in both planes, so the same maneuver of looking in both sagittal and transverse was essential. Furthermore, measurement of the aortic lumen, with overlying color Doppler confirming the true lumen, showed a uniform diameter (within a margin of error), as would be expected in young, healthy, asymptomatic patients (Figures 5 and 6). Most important to discerning fact from fiction, however, is understanding the normal anatomy neighbouring the abdominal aorta. The paired diaphragmatic crura are muscular fibers that originate on the lumbar spine and course anteriorly, creating a triangular retrocural space in which the abdominal aorta sits [8–9]. In this series of images, a trick of the “ultrasound eye” attempted to confuse the operators into thinking that the diaphragmatic crus that sits anterior to the abdominal aorta was a dissection flap inside of the vessel lumen (Figures 1-4, white star) instead of normal anatomy neighboring the abdominal aorta.

## Conclusion

POCUS users must be aware of the potential for both artifacts and normal anatomy that that mimic pathology. They must have enough understanding of both normal anatomy and probe maneuvers to distinguish pathology from artifact or mimic. Diaphragmatic crura can mimic an aortic dissection by confusing which structure is the true wall of the aorta. Both color Doppler and scanning the aorta in multiple planes can provide clarity to this confusion.
